# A Novel Scalable Reconfiguration Model for the Postdisaster Network Connectivity of Resilient Power Distribution Systems

**DOI:** 10.3390/s23031200

**Published:** 2023-01-20

**Authors:** Ahmed Imteaj, Vahid Akbari, Mohammad Hadi Amini

**Affiliations:** 1 School of Computing, Southern Illinois University, Carbondale, IL 62901, USA; 2 Nottingham University Business School, University of Nottingham, Jubilee Campus, Nottingham NG8 1BB, UK; 3 Knight Foundation School of Computing and Information Sciences, Florida International University, Miami, FL 33199, USA

**Keywords:** resilience, optimal power flow, power distribution network, network reconfiguration

## Abstract

The resilient operation of power distribution networks requires efficient optimization models to enable situational awareness. One of the pivotal tools to enhance resilience is a network reconfiguration to ensure secure and reliable energy delivery while minimizing the number of disconnected loads in outage conditions. Power outages are caused by natural hazards, e.g., hurricanes, or system malfunction, e.g., line failure due to aging. In this paper, we first propose a distribution-network optimal power flow formulation (DOPF) and define a new resilience evaluation indicator, the demand satisfaction rate (DSR). DSR is the rate of satisfied load demand in the reconfigured network over the load demand satisfied in the DOPF. Then, we propose a novel model to efficiently find the optimal network reconfiguration by deploying sectionalizing switches during line outages that maximize resilience indicators. Moreover, we analyze a multiobjective scenario to maximize the DSR and minimize the number of utilized sectionalizing switches, which provides an efficient reconfiguration model preventing additional costs associated with closing unutilized sectionalizing switches. We tested our model on a virtually generated 33-bus distribution network and a real 234-bus power distribution network, demonstrating how using the sectionalizing switches can increase power accessibility in outage conditions.

## 1. Introduction

Power systems can be considered to be the foundation of modern society, as they are essential for resilient energy delivery to society. Specifically, the resilient operation of power distribution networks against cyber–physical attacks and natural hazards plays a crucial role in the modern electrified world [1,2,3]. The redundant interruption and intrusion of power systems lead to power failures that can be caused due to extreme weather events, resource aging, digitalization, diversification, and artificial perturbations, resulting in unprecedented challenges. Frequent power outages indicate the ill-preparation of the power system during extreme events. For instance, in 2012, the US experienced Hurricane Sandy, which landed on the east coast of the United States and resulted in power outages for millions of people. In 2018, the deadliest wildfire was recorded in California, which burnt around 1,893,913 acres of land and damaged nearly 18,000 structures [4,5]. To avoid such losses and not wait passively, expecting such disasters to pass, a useful strategy is to ensure resilience within a system, which can be defined as the continuation of normal operation regardless of the occurrence of any unexpected events. Over the past few decades, natural and artificial catastrophes have damaged power infrastructures. In 2015, due to a major earthquake, a dozen hydropower plants were damaged in Nepal and encountered a total loss of 150 MW power of electricity, which directly impacted their national economy [6]. In addition, the 2004 tsunami that hit the coastal areas of Sri Lanka, Indonesia, India, and Thailand significantly damaged the national infrastructures and cost thousands of lives [7]. These events demonstrate that we need to emphasize developing effective mechanisms to leverage the resilience operations of power infrastructures, mitigate economic loss, and reduce people’s suffering during a disaster.

To improve the resilience of a power system, we need to reconfigure power distribution networks. Integrating multimodal sensors within each power grid could be effective in sensing environments. If any accidents or unwanted events occur, the power-grid could immediately pass the information to the server [8,9]. After receiving information from the affected power grid or its neighbor, the server could isolate the affected region without delay. The information exchange mechanism could also play a pivotal role during a natural disaster, particularly in a distributed power system. The power-grid agents’ network connectivity can help in formulating optimal power flow within distributed networks. The authors in [10] proposed dynamic network flow modeling to improve the resilience assessment of interdependent critical infrastructures. However, the proposed model did not cover the postdisaster network connectivity scenario and scalable reconfiguration of power distribution systems. The authors in [11] also developed a mixed-integer linear programming (MILP) framework on the basis of optimization formulations to generate the effect of tweaking transmission line reactance. They leveraged two different approaches of system resilience as a control mechanism of postdisturbance.

This study introduces a novel mixed-integer mathematical model to maximize the demand satisfaction rate (DSR) in a postdisaster situation of power distribution systems. The DSR for each node with a positive load demand is defined as the proportion of load demand of that node that is supplied in the reconfigured distribution network. We define the DSR of the entire power distribution network as the average of the achieved DSR values over all the nodes with load demand. Our metric was tailored towards the optimal deployment of sectionalizing switches and its evaluation using the demand satisfaction rate). There are other metrics that have been developed in the literature. In [12], a comprehensive review of resilience evaluation metrics for power distribution networks was provided. Hosseini and Parvania [13] developed specific resilience metrics such as expected load interruption rate and expected maximum load loss. In [14], the system recovery index [15] was used for the performance evaluation of a microgrid. In our model, some power lines in a power distribution network faced an outage due to a natural hazard such as an earthquake or hurricane. Such power outages impede load accessibility for a number of load–demand buses. Considering that, we optimized the use of sectionalizing switches to maximize power accessibility and develop an optimized model that reconfigures power distribution networks. Our proposed model optimally selects sectionalizing switches with their optimal directions for activation to maximize the achievable DSR given disruptions in power distribution networks.

We further analyze how to minimize the costs associated with using the sectionalizing switches, and suggest a constraint to maximize the load accessibility given the budget constraints. This is equivalent to a multiobjective optimization model in which the first objective is to maximize DSR, and the second is to minimize the number of utilized or closed sectionalizing switches. In order to produce the proposed optimization model, we used optimization solver ‘Gurobi optimizer’ with a free academic license. In summary, the contributions of this work are threefold:

- First, we define DSR as a quantified resilience measure and as the proportion of satisfied demand in the reconfigured network in a postdisaster condition.
- Second, we propose a model to maximize the obtained DSR in a reconfigured network given the sectionalizing switches. Our optimization model identifies the switches and the direction in which they should be used.
- We provide further analysis of our problem by adding cost constraints to investigate the multiobjective version in which the first objective is to maximize DSR while minimizing the overall costs.

The rest of this paper is organized as follows. In the next section, we provide the background literature of the studied problem. In Section 3, we first give the optimization model to minimize the load generation costs while satisfying the load demand of all the bus nodes. Then, we model the power distribution disturbance. In Section 4, we computationally test our optimization model on both a virtually generated power distribution network and a power distribution network from the real world.

## 2. Literature Review

Numerous optimization techniques have been developed to solve the optimal power flow (OPF) problem. Conventional techniques, such as nonlinear programming, quadratic programming, gradient’s method, and interior point methods, were employed in [16,17] and suffer from some downsides, e.g., local minima traps, dimensionality curses, and theoretical assumptions that do not guarantee an optimal global solution [17]. Adapting decentralized techniques and applying mathematical decomposition approaches to solve the OPF problem were applied, which are mostly based on microgrid operation based on decentralized algorithms on power management [18], power flow control [19], and simplified formulations of OPF [20,21,22]. The authors in [23] designed an algorithm on the basis of the alternating direction method of multipliers (ADMM) and moment relaxation that considered battery storage and used the decomposition technique to solve OPF problems. However, they did not formulate any multiphase OPF due to the complexity of the formulation. A generalization formulation on proximal dual consensus for constrained multiagent optimization was presented in [24] that could be applied to solve OPF-related problems. However, the authors did not consider intertemporal constraints, e.g., the state of charge, which could be effective for OPF. An OPF algorithm for the optimal operation of distribution grids was proposed by the authors in [25]. Though they were close to the fulfilment of microgrid operational requirements, they needed a multiperiod formulation that could be helpful in managing power storage. In [26], a teaching-learning-based strategy for optimized power flow was proposed, while in [27], a moth-swarm algorithm was presented. However, none of these works proposed situationally aware OPF algorithms to enhance resilience through reliable energy delivery in energy and transportation networks.

The authors in [28] introduced a synthetic model that could calculate the estimated time of restoration (ETR) for a load, generate a safe switching sequence, and reveal solutions for crews. Their proposed model could not perform system reconfiguration and was unable to make decisions regarding resource allocation. In [29], a distributed coordination scheme via information exchange among microgrid agents was presented. The authors discussed an approach to form microgrids on the basis of the local communication of the microgrid agents. Interesting work on leveraging the reconfiguration flexibility of distributed systems while considering the radiality constraints of microgrids was proposed in [30] and was specifically designed for postdisaster microgrid formation. The authors in [31] constructed an optimization model on the basis of inequitably distributed system restoration, and the model was effective in reconfiguring a system topology and dispatching optimal models to crews, even in the presence of unbalanced master power sources. Moreover, the authors in [32] established a spectral depiction of power distribution by capturing subtrees from different families within a transmission network. They proposed a strategy to localize the cascading failures within a power distribution network. However, they did not discuss how to perform characterization to reduce cascading failure by switching off the fault transmission lines.

Prior works also focused on restoring power distribution networks during disasters by adapting active islanding strategies, and considering distributed generators and microgrids [29,33,34,35,36,37]. For instance, spanning tree-based restoration algorithms were proposed to restore critical loads in [33,36]. However, the heuristic approach can be time-consuming while searching for a restoration plan. Few other works proposed partitioning the distributed system into smaller islands of grids and using distributed generators to restore loads [38,39]. However, these approaches considered single-phase distribution feeders without tie switches, rendering the methods inapplicable to the actual real-life problem of DSR. Some other related works [40,41,42] attempted to resolve the DSR problem by considering three-phase systems. These studies considered the practical parameters and models of the grid; for example, the authors in [42] considered an unbalanced distribution network and microgrids in the proposed service restoration algorithms. In this study, we focus on the topological properties of a distribution network graph and define a new resilience indicator, referred to as the demand satisfaction rate, to quantify the load loss. Using a novel mathematical model and an algorithm capable of matching optimal solutions rather than the physical constraints of the power grid, we focus on reconfiguring the graph topology using sectionalizing switches to enhance resilience on the basis of the identified indicator. In our prior works on power distribution networks, we developed abstract algorithms for optimal sensor placement [43] and outage detection [44] in power distribution networks on the basis of the topology of the grid. This class of studies mainly tackled the structural challenges that the power distribution grid may face, and complemented existing work with several layers of real-world details, including unbalanced three-phase models.

## 3. Optimal Power Flow

We modelled the distribution network as a graph G=(V,E) with *N* nodes, where *V* is the set of nodes and *E* is the set of links. Further, we denote the edge between nodes *i* and *k* with $l_{i,k}$; hence, $l_{i,k}\in E$ means there was a connection between these two nodes. Let $\delta_{i}$ denote the neighborhood of node *i*, i.e., $\delta_{i}=\left\{k\in V\;|\;l_{i,k}\in E\right\}$. We denote the impedance of connecting edge (power line/cable) between nodes *i* and *k* by $z_{ik}=r_{ik}+jx_{ik}$, where $r_{ik}$ is the resistance, and $x_{i,k}$ gives the reactance of link $l_{i,k}$. Then, $g_{ik}=\frac{r_{ik}}{x_{ik}^{2}+r_{ik}^{2}}$ and $b_{ik}=\frac{x_{ik}}{x_{ik}^{2}+r_{ik}^{2}}$ represent the conductance and susceptance of line $l_{i,k}$, respectively. We also assumed that we had *p* power generators in nodes indexed from 1 to *p*, where p<N, and we show this set via P={1,...,p}. In a feasible solution where the generated power is transferred to all the nodes in the distribution network, and all the nodes had access to sufficient power and assuming a quadratic cost function for power generation, the mathematical problem formulation can be given as in the following:

(1) $$\begin{array}{cc} \; & \min_{P_{G}}\sum_{n\in \delta_{G}}\left(a_{n}P_{G_{n}}^{2}+b_{n}P_{G_{n}}+c_{n}\right) \end{array}$$

(2) $$\begin{array}{cc} \; & P_{G_{i}}-P_{L_{i}}=\sum_{j\in \delta_{i}}\left[b_{ij}\left(\theta_{i}-\theta_{j}\right)+g_{ij}\left(\epsilon_{i}-\epsilon_{j}\right)\right],\;\forall i\in V \end{array}$$

(3) $$\begin{array}{cc} \; & \theta_{1}=0 \end{array}$$

(4) $$\begin{array}{cc} \; & {\underline{P}}_{G_{n}}\leq P_{G_{n}}\leq {\bar{P}}_{G_{n}},\;\forall n\in \delta_{p},\;\forall p\in P \end{array}$$

(5) $$\begin{array}{cc} \; & \underline{\epsilon_{i}}\leq \epsilon_{i}\leq \bar{\epsilon_{i}},\;\forall i\in V \end{array}$$

(6) $$\begin{array}{cc} \; & \underline{\theta_{i}}\leq \theta_{i}\leq \bar{\theta_{i}}\;\forall i\in V \end{array}$$

(7) $$\begin{array}{cc} \; & -{\bar{P}}_{ij}\leq \left[b_{ij}\left(\theta_{i}-\theta_{j}\right)+g_{ij}\left(\epsilon_{i}-\epsilon_{j}\right)\right]\leq {\bar{P}}_{ij},\;\forall (i,j)\in E\; \end{array}$$

where $\underline{\epsilon_{i}}=v_{{min}_{i}}-1$ and $\bar{\epsilon_{i}}=v_{{max}_{i}}-1$ denote the minimal and maximal lower bounds of voltage deviation, respectively. i=1 was taken to be the slack bus. Further, for the sake of consistency in the power balance equation, we assumed ${\bar{P}}_{G_{i}}={\underline{P}}_{G_{i}}=0$, $\forall i\notin \delta_{G}$. This ensures the elimination of the power injected via the transformer in the power balance equation. In order to ensure the feasibility of the power distribution, the constraint sets from (2) to (7) should hold. We only modelled active power flow because this study focuses on structural reconfiguration, considering the physical connectivity of power distribution lines. Our major focus is on the connectivity of the power grid after disasters and the outages of some lines. Hence, we considered the graph-based model of the grid, i.e., single diagram representation, and ensured an optimal reconfiguration strategy to maintain a maximal number of loads and minimize the disconnected loads. In addition to prior studies on cascading failure analysis, e.g., [32], in our prior works on optimal sensor placement [43,44], we made similar assumptions for the connectivity of the distribution grid, and only focused on the active power and operational topology of the network.

### 3.1. Disruption in the Power Network

As a result of a disruption in the distribution network, we assumed that a subset of links B⊂E were damaged, facing a line outage, and hence were not useable. In this scenario, the postdisruption distribution network is given by $G^{\prime }=\left(V,E\backslash B\right)$. This results in an unbalance in load access for some of the nodes in the network, and some might lose access to the power supply. Furthermore, a set of links, referred to as sectionalizing switches and denoted by *O*, only operate during such disruptions as emergency links. Assuming that all such links are active and operating after the incident, the graph of the distribution network changes into $G^{\prime \prime }=\left(V,E\cup O\backslash B\right)$. Even in this condition, the topology of the distribution network can prevent regular power supply to all the nodes.

Here, we define a problem in which we maximized the DSR over all the nodes in the network. Since there are some nodes without a load demand, by definition, DSR only calculates the average satisfied load demand over buses with a positive demand. We find the distribution flows of the power and the DSR for each bus. In this way, we can directly apply some restrictions, such as forcing a higher DSR for critical nodes such as hospitals and fire stations.

(8) $$\begin{array}{cc} \; & \mathrm{Max}\sum_{i\in V}\alpha_{i} \end{array}$$

(9) $$\begin{array}{cc} \; & P_{G_{i}}-P_{L_{i}}=\sum_{j\in \delta_{i}^{\prime \prime }}\left(f_{ij}-f_{ji}\right),\;\forall i\in V \end{array}$$

(10) $$\begin{array}{cc} \; & f_{ij}=\left[b_{ij}\left(\theta_{i}-\theta_{j}\right)+g_{ij}\left(\epsilon_{i}-\epsilon_{j}\right)\right],\;\forall \left(i,j\right)\in E\backslash \left\{O\cup B\right\} \end{array}$$

(11) $$\begin{array}{cc} \; & \left[b_{ij}\left(\theta_{i}-\theta_{j}\right)+g_{ij}\left(\epsilon_{i}-\epsilon_{j}\right)\right]\leq f_{ij}+\sum_{l\in V}P_{L_{l}}\left(1-y_{ij}\right)\;\forall \left(i,j\right)\in O \end{array}$$

(12) $$\begin{array}{cc} \; & -{\bar{P}}_{ij}\leq \left[b_{ij}\left(\theta_{i}-\theta_{j}\right)+g_{ij}\left(\epsilon_{i}-\epsilon_{j}\right)\right]\leq {\bar{P}}_{ij},\;\forall (i,j)\in E\cup O\backslash B \end{array}$$

(13) $$\begin{array}{cc} \; & y_{ij}+y_{ji}\leq 1,\;\forall \left(i,j\right)\in O \end{array}$$

(14) $$\begin{array}{cc} \; & f_{ij}\leq \sum_{l\in V}P_{L_{l}}*y_{ij}\;\forall \left(i,j\right)\in \left\{E\cup O\right\}\backslash B \end{array}$$

(15) $$\begin{array}{cc} \; & y_{ij}\in \left\{0,1\right\},\;\forall \left(i,j\right)\in \left\{E\cup O\right\}\backslash B \end{array}$$

(16) $$\begin{array}{cc} \; & f_{ij}\geq 0,\;\forall \left(i,j\right)\in \left\{E\cup O\right\}\backslash B \end{array}$$

We also enforce the constraints that are explained in (3)–(6). In the above formulation, the DSR value of each bus corresponds to the α value that is associated with that node. The objective function (8) maximizes the DSR values over all the nodes in the network. The $y_{ij}$ binary variables denote whether a link is used and in which direction it is operating. If a positive power flow is sent from bus *i* to bus *j* with the specified direction, $y_{ij}$ is 1; otherwise, it is 0. The $f_{ij}$ variables identify the amount of flow sent from node *i* to node *j*. The flow variable only identifies the positive amounts. Adding the sectionalizing switches, the structure of the network changes from a tree, and there are loops in the network, so we had to define the new flow variables to calculate the direction of the sent power. Constraint (9) is to set the flow balance equations similar to the one given in (2). The difference here is that the neighbors of each node are calculated in graph $G^{\prime \prime }=\left(V,E\cup O\backslash B\right)$ and are denoted by $\delta_{i}^{\prime \prime }$, and the flows are stored in the $f_{ij}$ variables. Then, on the basis of whether these flow variables are calculated on regular links or sectionalizing switches, the relationship of the flow variables and the characteristics of the power links is set on Constraints (10) and (11), respectively. Constraint Set (12) is the same as (7), with the difference that it is defined on the edge set of graph $G^{\prime \prime }$. Through Constraint Set (13), we identify the direction of the flow sent on each link and we calculate whether a sectionalizing switch is activated or not, and the direction in which the sectionalizing switch is used. Given Constraint (14), only positive flow is distributed in a link or sectionalizing switch in the direction in which it is activated. Lastly, we have all the restrictions on the variables.

## 4. Data Design and Performance Analysis

In this section, we test our model on two sets of instances. The first dataset was a virtually generated power distribution network with 33 nodes, and the second dataset was extracted from an actual power distribution network with 233 nodes. In both of these cases, we assumed that the cumulative amount of power generated in the source nodes was sufficient to satisfy the demand of the entire nodes. In this case, we could change the α parameters from a continuous variable between 0 and 1 into binary variables that took exactly the value of 1 if a node was reachable, and 0 if a node was not reachable from the source node.

### 4.1. Virtually Generated Power Distribution Network

**Figure 1:**
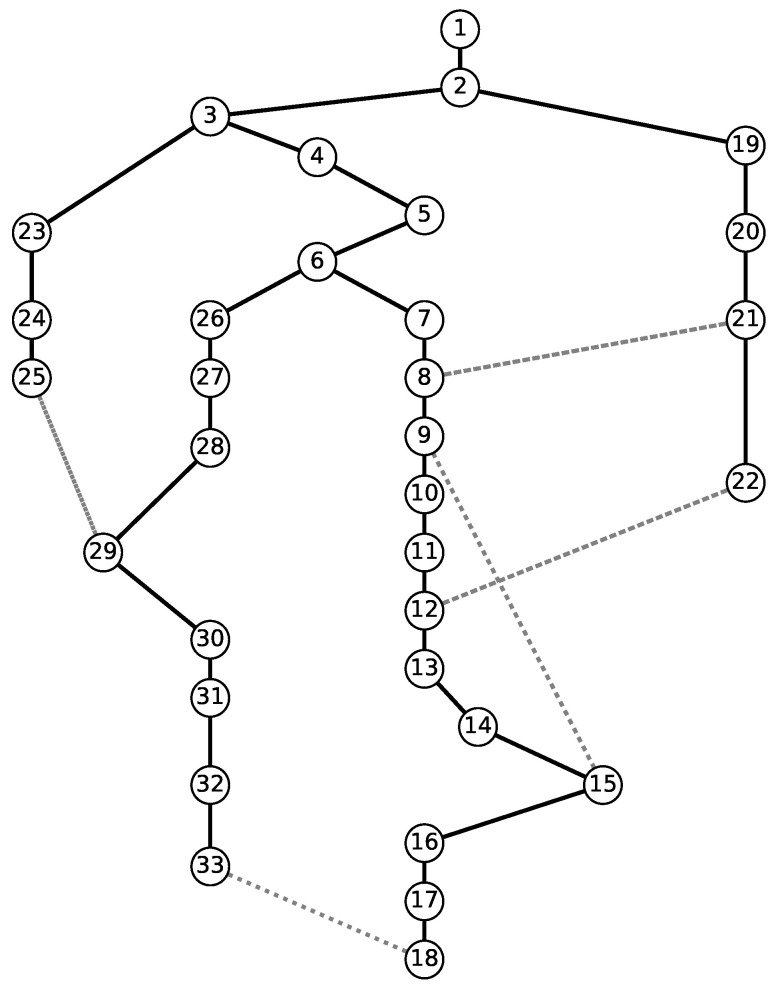
Graph representation of the Case 33 sample.

In this section, we tested our model on a virtually generated distribution network. This distribution network is given in Figure 1. In this network, the node indexed by 1 was the only node where power was generated and distributed. All the nodes other than this source node were assumed to have a positive demand. The links denoted by solid lines are the regular power lines, and the dashed lines are sectionalizing switches.

We generated 10 scenarios where damage to the links had occurred at different locations. Then, using sectionalizing switches, we maximized the number of nodes that had received their required load. As was stated above, we assumed the generated power in the source location to be sufficient to satisfy the load demand for all the nodes. These generated instances are given in Table 1.

In these instances, 32 nodes had positive demand, and Table 2 gives the result of testing our model for each of the cases given in Table 1. We first solved the problem in the case where the sectionalizing switches were not used, and then also solved the case where we could use as many sectionalizing switches as were available.

In this table, the first column on the left, shown by case, gives the corresponding case name from Table 1. The columns denoted by “Without Sectionalizing Switches” give the results of not using sectionalizing switches for the redistribution of the power, and the “With Sectionalizing Switches” columns give the results of testing the given model under the same disaster scenarios. “Number of Satisfied Nodes” gives the number of nodes that received their entire demand. We had 32 nodes that had a positive load demand. The next column, denoted by “Percentage of Satisfied Nodes”, gives the percentage of nodes that received their full demand. This is the value of the next column over 32. The next column shows the demand satisfaction rate, given that the total load demand was 37.15 units. Using the sectionalizing switches increased the achieved DSR in all the tested instances.

**Figure 2:**
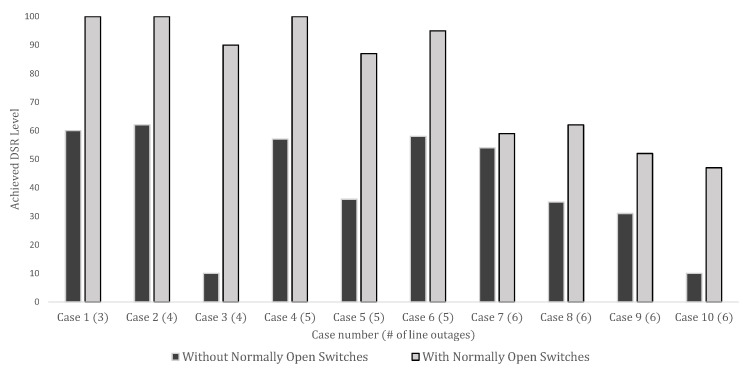
Comparison of DSR level with and without switches.

Figure 2 shows the relation of the achieved DSR levels with and without sectionalizing switches. While the achievable DSR without sectionalizing switches significantly depended on the location of the line outages, using the sectionalizing switches could significantly increase the achieved DSR in all the cases.

**Figure 3:**
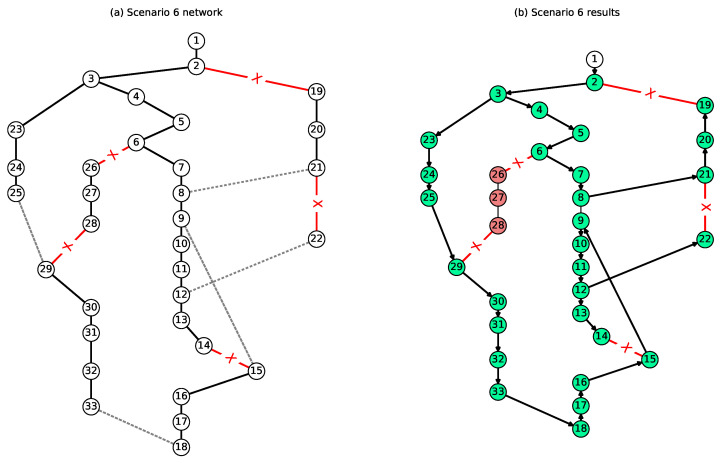
Obtained solution for Case C6.

Figure 1 gives the graphical representation of the virtual network, and Figure 3 gives the graphical representation of how the power was distributed given the line outages and sectionalizing switches of Case C6. In this case, links (2, 19), (6, 26), (28, 29), (14, 15), and (21, 22) experienced outages and could not be used. However, using the sectionalizing switches, we could satisfy the demand of up to 29 nodes, as given in the right-hand side of Figure 3, and Nodes 26–28 were not reachable with intact links or sectionalizing switches.

### 4.2. Real Power Distribution Network

In order to further study our optimization model and investigate its scalability, we generated 10 instances of a real power distribution network on the island of Rhodes, as shown in Table 3. This network has 234 buses and 239 lines. In these scenarios, on the basis of line outages and sectionalizing switches, the power distribution in the network changes. The goal of this model is to maximize the number of nodes that receive their full power demand or equivalently maximize the achieved DSR. In each of these scenarios, there were four lines that faced an outage in the hypothetical disaster situation. We tested each of these scenarios under three cases: (1) no sectionalizing switch is available; (2) three sectionalizing switches are available; (3) six sectionalizing switches are available. In the following, we first give the features of the tested instances in Table 3, and then demonstrate the results in Table 4.

Table 4 shows the results of testing our model on the 10 scenarios introduced in Table 3. In these instances, there were 88 nodes with a positive demand. In Table 4, we present the results of tested instances on a real network for 0, 3, and 6 sectioning switches considering the cases mentioned in Table 3. In Cases S3, S4, S6, and S8, using only 3 sectionalizing switches did not help in increasing the achieved DSR; however, by using 6 sectionalizing switches, we were able to significantly increase the achieved DSR values. On the other hand, in Scenarios S1, S2, and S10, the increase in the gained DSR in cases with 3 and 6 sectionalizing switches was not significant. For the rest of the scenarios, including S5, S7, and S9, an increasing trend in the achieved DSR from the cases with 0, 3, and 6 sectionalizing switches was evident. Another important observation regards the scalability of the proposed mathematical model and the optimization problem. In all of these problem instances, the model was solved within only a few seconds. Given that the instances with 234 buses and 239 lines were solved in merely a few seconds, we could confirm the performance of the optimization model and that it could solve even larger real-world instances in under a minute.

### 4.3. Sensitivity Analysis of the Number of Sectionalizing Switches

In the formulation presented in Section 3.1, we did not set a restriction on the number of sectionalizing switches, and only maximized the DSR. However, since there is a cost associated with using these sectionalizing switches, the number of used sectionalizing switches should be kept at a minimal level. For that, we could take different approaches. One approach is to set an upper limit on the number of sectionalizing switches that are used. Another approach is to use a multiobjective function that first maximizes the DSR and then minimizes the number of used sectionalizing switches. Here, we further investigate the scenarios for the real power distribution network introduced above to analyze what level of DSR can be achieved with an upper limit on the number of used sectionalizing switches. We obtained the maximal DSR level for the six available sectionalizing switches from Table 4. For example, for Scenario S6, using all the introduced sectionalizing switches, the maximal achievable DSR level was 89%. To perform this analysis, we defined an upper bound on the number of used sectionalizing switches, and added this constraint as

$$\sum_{(i,j)\in O}y_{ij}+y_{ji}\leq U$$

to the model given in Section 3.1, where *U* gives the imposed upper limit. The results of testing these scenarios are given in Table 5. As observed, we did not need to open all six switches to achieve the maximal DSR level in any of these instances. For Scenario S4, only opening 1 sectionalizing switch was sufficient to achieve the maximal possible DSR level; for Scenarios S1, S3, and S5, only two sectionalizing switches were needed to guarantee the maximal DSR level, and for Scenarios S2, and S6–S9, using three sectionalizing switches that had been carefully chosen resulted in the maximal achievable DSR level. Among these scenarios, only in Scenario S9 did we need to utilize more than 3 sectionalizing switches to achieve the maximal possible DSR. For this scenario, opening 4 normally open switches obtained the maximal achievable DSR.

**Figure 4:**
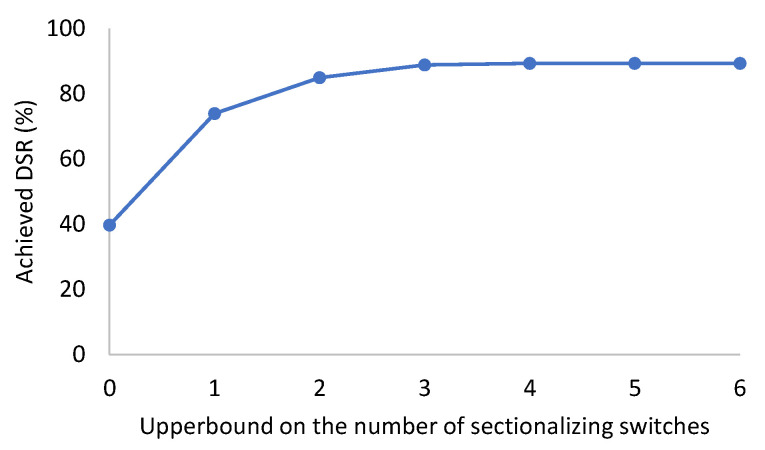
Average achieved DSR with limit on utilized sectionalizing switches.

Figure 4 gives the average results of testing our model on the 10 generated instances for the real power distribution network. This graph shows how using more sectionalizing switches can enable us to achieve a higher DSR. For example, over these instances, on average, if no sectionalizing switches were used, only just under 40% of the load demand could be satisfied. This value increased to almost 74% by using only one sectionalizing switch. The average DSR value increased to 85 and 89% once there was an upper limit of 2 and 3 on the utilized sectionalizing switches, respectively. The achieved DSR, however, remained just over 89% once the number of utilized sectionalizing switches had increased beyond 3. This confirmed the importance of the optimal selection of these switches to also consider the economic aspect of this reconfiguration while maximizing the achievable load demand.

## 5. Conclusions

In the immediate stage after a disaster, some lines in power distribution networks face an outage. This outage on the lines impedes accessibility to some of the buses in the power distribution network. In this study, we addressed the use of sectionalizing switches to increase power accessibility in postdisruption situations, tailoring a graph-based distribution network model using a single-line diagram. We defined the demand satisfaction rate (DSR) concept and developed a mixed-integer programming model to show how the use of sectionalizing switches can significantly increase DSR. Overall, the outcomes of this research were as follows:

1. Our proposed mathematical model could choose the sectionalizing switches and set the direction to maximize the level of DSR.
2. The performance evaluation of our proposed model considering real-world settings with 234 buses and 239 lines demonstrates that our proposed model is scalable even in large-scale power distribution networks.
3. Our investigations also showed that maximizing the level of DSR does not necessarily mean maximizing the expenditures on sectionalizing switch facilities. For example, in some scenarios where six sectionalizing switches were available, using only one sectionalizing switch increased the DSR value to almost 74%, which was under 40% if no sectionalizing switch was used.

In future work, we plan to consider fault isolation as an effective protection scheme to minimize the impact of line failure after natural hazards.

## Figures and Tables

**Table 1:** Scenarios for the virtual distribution network.

Case	Sectionalizing Switches	Line Outages
C1	(9, 15), (12, 22), (18, 33), (25, 29), (8, 21)	(10, 11), (28, 29), (20, 21)
C2	(9, 15), (12, 22), (18, 33), (25, 29), (8, 21)	(3, 23), (13, 14), (21, 22), (16, 17)
C3	(9, 15), (12, 22), (18, 33), (25, 29), (8, 21)	(2, 3), (10, 11), (14, 15), (21, 22)
C4	(9, 15), (12, 22), (18, 33), (25, 29), (8, 21)	(10, 11), (28, 29), (20, 21), (8, 9), (13, 14)
C5	(9, 15), (12, 22), (18, 33), (25, 29), (8, 21)	(31, 32), (15, 16), (6, 7), (21, 22), (3, 23)
C6	(9, 15), (12, 22), (18, 33), (25, 29), (8, 21)	(28, 29), (14, 15), (6, 26), (2, 19), (21, 22)
C7	(9, 15), (12, 22), (18, 33), (25, 29), (8, 21)	(28, 29), (20, 21), (24, 25), (26, 27), (19, 20), (16, 17)
C8	(9, 15), (12, 22), (18, 33), (25, 29), (8, 21)	(10, 11), (6, 26), (30, 29), (23, 24), (13, 14), (21, 22)
C9	(9, 15), (12, 22), (18, 33), (25, 29), (8, 21)	(9, 10), (28, 29), (2, 19), (32, 33), (15, 16), (23, 24)
C10	(9, 15), (12, 22), (18, 33), (25, 29), (8, 21)	(2, 3), (10, 11), (14, 15), (21, 22), (6, 7), (29, 30)

**Table 2:** Results of the tested instances on the virtual network.

	Without Sectionalizing Switches			With Sectionalizing Switches		
**Case**	**Number of Satisfied Nodes**	**Percentage of Satisfied Nodes**	**Achieved DSR**	**Number of Satisfied Nodes**	**Percentage of Satisfied Nodes**	**Achieved DSR**
C1	17	53%	60%	32	100%	100%
C2	23	72%	62%	32	100%	100%
C3	4	13%	10%	27	84%	90%
C4	15	47%	57%	32	100%	100%
C5	14	44%	36%	27	84%	87%
C6	15	50%	58%	29	90%	95%
C7	19	59%	54%	21	65%	59%
C8	13	40%	35%	22	69%	62%
C9	12	38%	31%	22	69%	52%
C10	4	13%	10%	16	50%	47%

**Table 3:** Scenarios.

	Sectionalizing Switches	Line Outages
S1-1	{(6, 15), (64, 154), (104, 109)}	{(98,100),(139, 141), (117,119), (215, 217)}
S2-1	{(6, 15), (64, 154), (104, 109)}	{(117,119), (215, 217), (95, 109), (135, 138)}
S3-1	{(6, 15), (64, 154), (104, 109)}	{(89,93), (112,113), (95, 96), (46, 50)}
S4-1	{(6, 15), (64, 154), (104, 109)}	{(112, 113), (154, 157), (54, 68), (42, 44)}
S5-1	{(6, 15), (64, 154), (104, 109)}	{(127, 129), (69, 71), (129, 131), (95, 96)}
S6-1	{(6, 15), (64, 154), (104, 109)}	{(25,28),(60,62),(98,100),(115, 117)}
S7-1	{(6, 15), (64, 154), (104, 109)}	{(95,187), (135,138), (89, 93), (95,96)}
S8-1	{(6, 15), (64, 154), (104, 109)}	{(95,109), (52, 54), (152, 154), (113, 115)}
S9-1	{(6, 15), (64, 154), (104, 109)}	{(8,14), (138, 148), (127, 129), (96, 98)}
S10-1	{(6, 15), (64, 154), (104, 109)}	{(113,115), (138, 148), (187, 95), (76,77)}
S1-2	{(6, 15), (3, 135), (64, 154), (54, 80), (104, 109), (57, 14)}	{(98,100),(139, 141), (117,119), (215, 217)}
S2-2	{(6, 15), (3, 135), (64, 154), (54, 80), (104, 109), (57, 14)}	{(117,119), (215, 217), (95, 109), (135, 138)}
S3-2	{(6, 15), (3, 135), (64, 154), (54, 80), (104, 109), (57, 14)}	{(89,93), (112,113), (95, 96), (46, 50)}
S4-2	{(6, 15), (3, 135), (64, 154), (54, 80), (104, 109), (57, 14)}	{(112, 113), (154, 157), (54, 68), (42, 44)}
S5-2	{(6, 15), (3, 135), (64, 154), (54, 80), (104, 109), (57, 14)}	{(127, 129), (69, 71), (129, 131), (95, 96)}
S6-2	{(6, 15), (3, 135), (64, 154), (54, 80), (104, 109), (57, 14)}	{(25,28),(60,62),(98,100),(115, 117)}
S7-2	{(6, 15), (3, 135), (64, 154), (54, 80), (104, 109), (57, 14)}	{(95,187), (135,138), (89, 93), (95,96)}
S8-2	{(6, 15), (3, 135), (64, 154), (54, 80), (104, 109), (57, 14)}	{(95,109), (52, 54), (152, 154), (113, 115)}
S9-2	{(6, 15), (3, 135), (64, 154), (54, 80), (104, 109), (57, 14)}	{(8,14), (138, 148), (127, 129), (96, 98)}
S10-2	{(6, 15), (3, 135), (64, 154), (54, 80), (104, 109), (57, 14)}	{(113,115), (138, 148), (187, 95), (76,77)}

**Table 4:** Results of the tested instances on a real network.

	Without Sectionalizing Switches		3 Sectionalizing Switches		6 Sectionalizing Switches	
**Case**	**Number of Satisfied Nodes**	**Achieved DSR**	**Number of Satisfied Nodes**	**Achieved DSR**	**Number of Satisfied Nodes**	**Achieved DSR**
S1	58	66%	80	85%	80	85%
S2	56	60%	79	85%	84	89%
S3	23	30%	23	30%	81	84%
S4	20	28%	20	28%	76	78%
S5	34	40%	55	60%	88	100%
S6	12	20%	12	20%	83	89%
S7	46	52%	61	68%	88	100%
S8	25	31%	25	31%	82	84%
S9	5	7.7%	77	90%	88	100%
S10	56	62%	75	78%	82	84%

**Table 5:** Sensitivity analysis of the number of sectionalizing switches.

Upper Limit	1		2		3	
**Case**	**Number of Satisfied Nodes**	**Achieved DSR**	**Number of Satisfied Nodes**	**Achieved DSR**	**Number of Satisfied Nodes**	**Achieved DSR**
S1	74	80%	80	85%	80	85%
S2	71	75%	79	86%	84	89%
S3	58	61%	81	84%	81	84%
S4	76	78%	76	78%	76	78%
S5	67	80%	88	100%	88	100%
S6	74	81%	80	85%	83	89%
S7	64	78%	79	93%	88	100%
S8	56	60%	75	76%	82	84%
S9	60	75%	71	85%	81	95%
S10	66	71%	75	77%	82	84%

## Data Availability

The original contributions presented in the study are included in the article, further inquiries can be directed to the corresponding author.

## Abbreviations

The following abbreviations are used in this manuscript:

DSR	Demand satisfaction rate
DOPF	Distribution network optimal power flow formulation
ADMM	Alternating direction method of multipliers
OPF	Optimal power flow
DRL	Deep reinforcement learning
